# 

**Published:** 1890-07-26

**Authors:** 


					^RlCE ONE PENNY.
-July 26th, 1890.
trans**; . lPe W^eral Post Office as a Newspaper for
ission m the United Kingdom and Abroad,]
WITH EXTRA NURSING SUPPLEMENT.
Per Annum, 6s. 6d.j post free.
Monthly Parts, 6<L; post free 8d.
Ditto per Annum, 8s.. post free.
SATURDAY, JULY 26th, 1890

				

